# Different Adjuvants Induce Common Innate Pathways That Are Associated with Enhanced Adaptive Responses against a Model Antigen in Humans

**DOI:** 10.3389/fimmu.2017.00943

**Published:** 2017-08-14

**Authors:** Wivine Burny, Andrea Callegaro, Viviane Bechtold, Frédéric Clement, Sophie Delhaye, Laurence Fissette, Michel Janssens, Geert Leroux-Roels, Arnaud Marchant, Robert A. van den Berg, Nathalie Garçon, Robbert van der Most, Arnaud M. Didierlaurent, On Behalf of the ECR-002 Study Group, Viviane Bechtold

**Affiliations:** ^1^GSK, Rixensart, Belgium; ^2^Center for Vaccinology, Ghent University, Ghent University Hospital, Ghent, Belgium; ^3^Freelance, Jodoigne, Belgium; ^4^Institute for Medical Immunology, Université Libre de Bruxelles, Gosselies, Belgium; ^5^BIOASTER, Lyon, France

**Keywords:** vaccine adjuvants, AS01, AS03, AS04, innate immune response, adaptive immune response, interferon-γ

## Abstract

To elucidate the role of innate responses in vaccine immunogenicity, we compared early responses to hepatitis B virus (HBV) surface antigen (HBsAg) combined with different Adjuvant Systems (AS) in healthy HBV-naïve adults, and included these parameters in multi-parametric models of adaptive responses. A total of 291 participants aged 18–45 years were randomized 1:1:1:1:1 to receive HBsAg with AS01_B_, AS01_E_, AS03, AS04, or Alum/Al(OH)_3_ at days 0 and 30 (ClinicalTrials.gov: NCT00805389). Blood protein, cellular, and mRNA innate responses were assessed at early time-points and up to 7 days after vaccination, and used with reactogenicity symptoms in linear regression analyses evaluating their correlation with HBs-specific CD4^+^ T-cell and antibody responses at day 44. All AS induced transient innate responses, including interleukin (IL)-6 and C-reactive protein (CRP), mostly peaking at 24 h post-vaccination and subsiding to baseline within 1–3 days. After the second but not the first injection, median interferon (IFN)-γ levels were increased in the AS01_B_ group, and IFN-γ-inducible protein-10 levels and IFN-inducible genes upregulated in the AS01 and AS03 groups. No distinct marker or signature was specific to one particular AS. Innate profiles were comparable between AS01_B_, AS01_E_, and AS03 groups, and between AS04 and Alum groups. AS group rankings within adaptive and innate response levels and reactogenicity prevalence were similar (AS01_B_ ≥ AS01_E_ > AS03 > AS04 > Alum), suggesting an association between magnitudes of inflammatory and vaccine responses. Modeling revealed associations between adaptive responses and specific traits of the innate response post-dose 2 (activation of the IFN-signaling pathway, CRP and IL-6 responses). In conclusion, the ability of AS01 and AS03 to enhance adaptive responses to co-administered HBsAg is likely linked to their capacity to activate innate immunity, particularly the IFN-signaling pathway.

## Introduction

Upon detecting conserved pathogen- or danger-associated molecular patterns, the innate immune system mediates rapid and multifaceted responses composed of cellular and soluble components. These responses are activated *via* engagement of pattern recognition receptors (PRRs) expressed on several immune and non-immune cell types. Though the mechanisms involved have yet to be demonstrated in humans, innate immunity is thought to control the magnitude and quality of adaptive immune responses. The complex interactions between these two arms of the immune system have not completely been unraveled, and their distinction with respect to memory features is blurred by findings of innate-like T cells, as well as innate cells [macrophages, natural killer (NK) cells, monocytes] displaying epigenetic changes following activation (“trained immunity”) (1–5). Vaccine adjuvants have been demonstrated to activate receptors and pathways that modulate the innate response (6), rendering adjuvanted vaccines attractive tools to study the interplay between innate and adaptive immune systems in humans. The mechanisms of action of the innate pathways triggered by many human vaccine adjuvants are not fully delineated, and adjuvant development to date has largely focused on the use of toll-like receptor ligand family of PRRs (7, 8).

Adjuvant Systems (AS) AS01, AS03, and AS04 combine different stimulants of innate immunity. They were developed with the aim to augment vaccine antigen-specific T-cell and antibody responses (9, 10), and selected for use in several candidate or licensed vaccines. Their immuno-enhancing capacities and the acceptable safety profiles of vaccines containing these AS have been demonstrated in myriad clinical trials [reviewed in Ref. (9, 11, 12)]. AS01, combining two immunostimulants [TLR4 ligand 3-*O*-desacyl-4′-monophosphoryl lipid A (MPL) and the purified saponin QS-21] in a liposome-based formulation, is used in candidate vaccines against malaria (RTS,S) and herpes zoster (HZ/su), for which vaccine efficacy was demonstrated in phase-III trials (13–15). AS01 is also used in candidate vaccines against tuberculosis and human immunodeficiency virus (16, 17). AS03, containing α-tocopherol and squalene in an oil-in-water (o/w) emulsion, is used in several influenza vaccines, i.e., trivalent inactivated and A(H1N1)pdm09 influenza vaccines, H5N1 pre-pandemic influenza vaccines, and candidate H7N1 and H7N9 pandemic influenza vaccines (18–22). AS04, containing MPL adsorbed on aluminum salt (AlPO_4_) is used in a licensed human papillomavirus (HPV)-16/18 vaccine and a licensed hepatitis B virus (HBV) vaccine used for hemodialized patients (23, 24).

The main objectives of the respective clinical trials of AS-adjuvanted vaccines included evaluation of vaccine safety and reactogenicity, and of immunogenicity in terms of the magnitude of adaptive responses specific for the vaccine antigens. To support these evaluations, a solid understanding of the mode of action of adjuvanted vaccines, including the interplay between early inflammatory and adaptive responses, is crucial. Thus far, evaluations performed in animal models demonstrated that AS01, AS03, and AS04 directly affect innate immune cell populations and effectors. In mice, all AS potentiated transient inflammatory responses at both the injection site (muscle) and the draining lymph node (dLN), resulting in increased numbers of activated antigen-presenting dendritic cells in the dLN, and sequential stimulation of adaptive responses (25–27). Transient systemic responses of the acute-phase response marker C-reactive protein (CRP), cytokines, and changes in neutrophil, monocyte and/or eosinophil blood fractions were also observed in animal models (25–30). Yet, the innate immunity promoted by AS-adjuvanted vaccines in humans has not been characterized nor compared between different formulations, with the exception of the early cytokine responses described for an AS04-adjuvanted HPV-16/18 vaccine (31).

Previously, we reported head-to-head comparisons of the safety and adaptive responses for adjuvanted vaccines containing the prototypic HBV surface antigen (HBsAg) in young, HBV-naïve adults (32–34), the most recent of which compared formulations adjuvanted with AS01_B_, AS01_E_ (half-dose AS01_B_ with respect to MPL and QS-21 quantities), AS03, AS04 or aluminum salt (Al(OH)_3_; Alum) (34). From this study, a pattern emerged in which HBs-specific CD4^+^ T-cell and antibody responses could be generally ranked, in order of decreasing magnitudes, from AS01, to AS03, to AS04, to Alum. Since a similar ranking seemed to apply to the prevalence of solicited adverse events (AEs), which may reflect inflammatory signals, we hypothesized that the adaptive and early inflammatory (innate) responses to these vaccines could be correlated. To evaluate this hypothesis, we characterized innate vaccine responses at the protein, gene expression, and cellular level in peripheral blood, in order to use these data in linear regression models of the adaptive responses. The modeling of gene expression data was performed on a limited but robust dataset generated by qPCR. This was done in order to provide sufficient power to the model and to enhance the likelihood of obtaining meaningful results in this hypothesis-driven study. Innate responses were summarized using principal component (PC) analysis, which allowed dissecting out the complex mix of early innate variables and identifying innate signatures governing these associations.

In this first-time comparison of blood innate responses to AS01, AS03, and AS04 in humans, we observed rapid (starting at 3–6 h) yet transient changes in blood innate parameters, some of which were shown to correlate with the magnitude of the adaptive responses.

## Materials and Methods

### Study Design

The randomized, controlled phase II trial (ClinicalTrials.gov: NCT00805389) was performed at 14 study centers (34). The protocol was approved by all institutional Ethics Committees and conducted in accordance with the Helsinki Declaration and Good Clinical Practice guidelines. Written informed consent was obtained from each participant before trial participation. Participants were healthy HBV-naïve men or women 18–45 years of age who received two intramuscular injections of vaccine containing HBsAg (20 µg dose) adjuvanted with AS01_B_, AS01_E_, AS03_A_, AS04 (*FENDrix*), or Alum (*Engerix-B*) at days 0 and 30. Participants were followed up to day 360. Safety and reactogenicity up to day 60 in the total vaccinated cohort, and adaptive responses in the according-to-protocol (ATP) cohort for adaptive immunogenicity up to day 60 were described previously (34).

One of the study’s secondary endpoints, innate immunogenicity, was evaluated for the ATP cohort for innate immunogenicity, which included all participants not meeting elimination criteria during the study and for whom innate immunogenicity data were available (Table 1). A targeted 375 participants were randomized 1:1:1:1:1 using Internet-based block randomization (SASv8.2; SAS Institute Inc.). Two datasets were generated and modeled separately: one comprising data of clinical laboratory parameters (hematology, CRP) and serum cytokine and chemokine levels generated for the full ATP cohort (“clinical laboratory/serum dataset”) and a second set containing quantitative polymerase chain reaction (qPCR) data for a subset of the cohort, including only participants from the ImmuneHealth (Gosselies, Belgium) study center (“qPCR dataset”). Modeling was performed using a subset of the ATP cohorts (see Statistical Analyses below).

**Table 1:** Cohorts and subsets.

Cohorts or subsets (evaluations)		Group (*n*)					Total
		AS01_B_	AS01_E_	AS03	AS04	Alum	(*N*)
Total vaccinated^a^	(safety, reactogenicity)	143	142	141	145	142	713
ATP, adaptive immunogenicity up to D60^a^	(T-cell response^b^)	121	120	118	124	116	599
ATP, adaptive immunogenicity up to D60^a^	(antibody response^c^)	59	57	58	62	57	293
ATP, innate immunogenicity	(innate immunity^c^)	59	57	59	61	55	291
• Clinical laboratory/serum dataset		59	57	59	61	55	291
• qPCR subset		18	23	28	22	21	112
ATP, innate/adaptive immunogenicity^d^	(T cell/antibody response^d^)	59	56	57	61	53	286
Modeling subset:^e^	(multi-parametric analyses)	53	53	52	51	47	256
• Clinical laboratory/serum dataset		53	53	52	51	47	256
• qPCR dataset		11	18	23	15	17	84

*ATP, according-to-protocol*.

*^a^Cohorts and evaluations described in Ref. (34)*.

*^b^HBs-specific CD4^+^ T-cell frequencies at day 44 was the primary endpoint of the study*.

*^c^Anti-HBs antibody concentrations at day 60 and innate immune responses were among the secondary endpoints of the study. For qPCR assays 146 subjects were randomized in the ImmuneHealth (Gosselies, Belgium) study center, of whom 112 were included in the ATP cohort and had at least one qPCR result available which resulted in slightly uneven group sizes*.

*^d^For the current report, T-cell and antibody responses were characterized for the ATP cohort for innate immunogenicity, excluding the five participants who were previously excluded from the ATP cohort for adaptive immunogenicity, for reasons described in Ref. (34)*.

*^e^The modeling subset included only subjects with data available for each parameter in the model (among T-cell and antibody responses, reactogenicity scores, and innate clinical laboratory/serum or qPCR data)*.

### Adjuvants

One AS01_B_ dose contained 50 µg MPL (3-*O*-desacyl-4′-monophosphoryl lipid A), 50 µg QS-21 (*Quillaja saponaria* Molina, fraction 21; licensed by GSK from Antigenics Inc., a wholly owned subsidiary of Agenus Inc., a Delaware, USA corporation) and liposomes. One AS01_E_ dose contained 25 µg each of MPL and QS-21 and liposomes. One dose of AS03_A_ (elsewhere in this article referred to as AS03) contained 11.86 mg α-tocopherol and squalene in an o/w emulsion. One AS04 dose contained 50 µg MPL adsorbed on Al salt (500 µg Al^3+^ in the form of AlPO_4_). One dose of Alum contained 500 µg Al^3+^ in the form of Al(OH)_3_.

### Innate Response Evaluations

Blood samples for innate response evaluations were collected before vaccination (days 0 and 30), 3–6 h, 1 day and, for qPCR analysis only, 14 days after dose 1 (3–6 h, day 1 and day 14), and 3–6 h, 1, 3, and 7 days after dose 2 (3–6 h on day 30, day 31, day 33, and day 37, respectively).

#### Cytokines

Cytokine concentrations in serum were measured using cytometric bead array (CBA) commercial kits, i.e., BD CBA Human Enhanced Sensitivity Master Buffer kits [for interleukin (IL)-1β, IL-6, IL-5, IL-10, tumor necrosis factor (TNF)-α, and interferon (IFN)-γ] and BD CBA Human Flex Set kits [for IFN-γ-inducible protein (IP)-10 and monocyte chemoattractant protein (MCP)-1], according to the manufacturer’s instructions. Since these kits were not validated, qualification was performed internally to establish their cutoff values, which were subsequently set at 0.822 pg/mL for IL-1β, IL-6, TNF-α, IL-5, and IL-10, 40 pg/mL for IP-10 and MCP-1, and 7.407 pg/mL for IFN-γ. Of note, the latter cutoff for IFN-γ was higher than the limit of quantitation of the IFN-γ ELISA used in a recent study, i.e., 1.0 pg/mL (35). Concentrations below these assay cutoffs were given an arbitrary value of one-half of the cutoff value.

#### Hematology and CRP

Blood samples for hematology assessment were analyzed within 24 h after collection using a standard hematology analyzer. Serum CRP concentrations were measured and counts of white blood cells (WBC: lymphocytes, monocytes, eosinophils, basophils, neutrophils) recorded. Tests were conducted by ISO 15189-accredited labs. As this was a multicentric study, a set of normal ranges was provided by each study center. In order to compute summary statistics, results were first normalized. To facilitate interpretation, normalization was done using reference ranges from one center (ImmuneHealth), as follows: normalized data = *Ls* + (*x*−*Lx*) × [(*Us*−*Ls*)/(*Ux*−*Lx*)], where *x* = raw data; *Ux*/*Lx* = upper/lower normal limit of the local normal range applicable to *x*; *Us*/*Ls* = upper/lower normal limit of the corresponding reference range. For differential cell counts, this formula was only applied to subjects from the centers expressing their results in absolute counts (i.e., in the specific unit as was used in the reference center), for whom the results are shown here.

#### Gene Expression

Expression of 14 target genes encoding cytokines or transcription factors implicated in the innate response (listed in Table S1 in Supplementary Material) was assessed by qPCR. Total RNA was isolated from whole blood collected in PAXgene Blood RNA tubes (PreAnalytiX) using the RNeasy RNA purification kit and the BioRobot MDx system (both Qiagen, Valencia, CA, USA), according to the manufacturer’s guidelines. RNA integrity numbers (RINs) were determined using the Agilent 2100 Bioanalyzer and expert software (Agilent Technologies, Palo Alto, CA, USA). RNA with RIN > 7 was included in the analysis. The 260/280 ratio was measured using an ND-1000 spectrophotometer (NanoDrop Technologies, Wilmington, DE, USA), and only RNA samples with a 260/280 ratio ≥1.7 were used in the analysis. Reverse transcription was performed using a high-capacity cDNA Reverse Transcription Kit (Applied Biosystems) with random primers and MultiScribe reverse transcriptase (Applied Biosystems). Transcription levels of the target genes and housekeeping genes (HKG) *PPIB, DECR1*, and *GUSB* were measured by qPCR using TaqMan low-density array cards (TLDAs; Applied Biosystems, Foster City, CA, USA). TaqMan assay IDs were as follows: *PPIB*, HS00168719; *DECR1*, Hs00154728_m1; *GUSB*, Hs99999908_m1; *IL1B*, Hs01555410_m1; *PTGS2/COX2*, Hs0153133_m1; *MKP1/DUSP1*, Hs00610256_g1; *NFATc2*, Hs00905451_m1; *TNFA*, Hs99999043_m1; *TNFRSF9*/*4-1BB*, Hs00155512_m1; *FAS*/*TNFRSF6*, Hs00169544_m1; *IFNG*, Hs00174142_m1; *STAT1*, Hs0019544_m1; *IRF1*, Hs01013996_m1; *MX1*, Hs00895608_m1; *IL12A*, Hs01073447_m1; *Ki67*/*MKI67*, Hs01032443_m1; *CXCL10*, Hs00171042_m1; 18s (endogenous control; Applied Biosystems), HS99999901_s1. Each qPCR reaction was qualified and validated for efficiency, linearity, and precision in a specific range of threshold cycle (Ct) values. For values higher than LOQ (Ct = 32), the value has been replaced by (LOQ + 1). Geometric means of the Ct of the HKG and means of the Ct for each duplicated target gene were calculated, with the following normalization for each target gene: ΔCt = geomean Ct_HKG_−mean Ct_target gene_. Impact of the treatments on mRNA levels was expressed in ΔΔCt values representing the relative quantification of the ΔCt value at a given post-vaccination time-point over the ΔCt at pre-vaccination (days 0 and 30) by calculating ΔΔCt = ΔCt post − ΔCt pre. Fold changes (FCs) were calculated as 2^ΔΔCt^. Genes with FC > |2.0| were considered differentially expressed.

### Statistical Analyses

#### ATP Cohort Descriptions

Descriptive statistical analyses were conducted using SAS v9. Characterization of the three model parameters was performed for the ATP cohort for innate immunogenicity (for innate responses and reactogenicity evaluations) and for the same ATP cohort excluding the participants who were previously (34) excluded from the ATP cohort for adaptive immunogenicity (for adaptive responses; Table 1). Given the different laboratory quantitation standards, responses of the innate variables were expressed in FCs over their pre-vaccination baselines (days 0 and 30). The median FCs were visualized in heat maps generated in R (https://www.r-project.org/), in which data were zero-mirrored for symmetrical presentation of over- and under-expression, as follows: FC_A,B_ = [A/B] if A ≥ B, or FC_A,B_ = [−B/A], if A < B, (where A and B are post- and pre-vaccination responses). Significant differences in post-vaccination IL-6 and IP-10 levels between an AS group and the Alum group were assessed using a testing cascade with α-recycling (36) (starting with a Kruskal–Wallis rank sum group test and cascading into Wilcoxon rank sum tests), and overall statistical significance level α of 0.05.

#### Datasets Multi-Parametric Analyses

**Figure 1:**
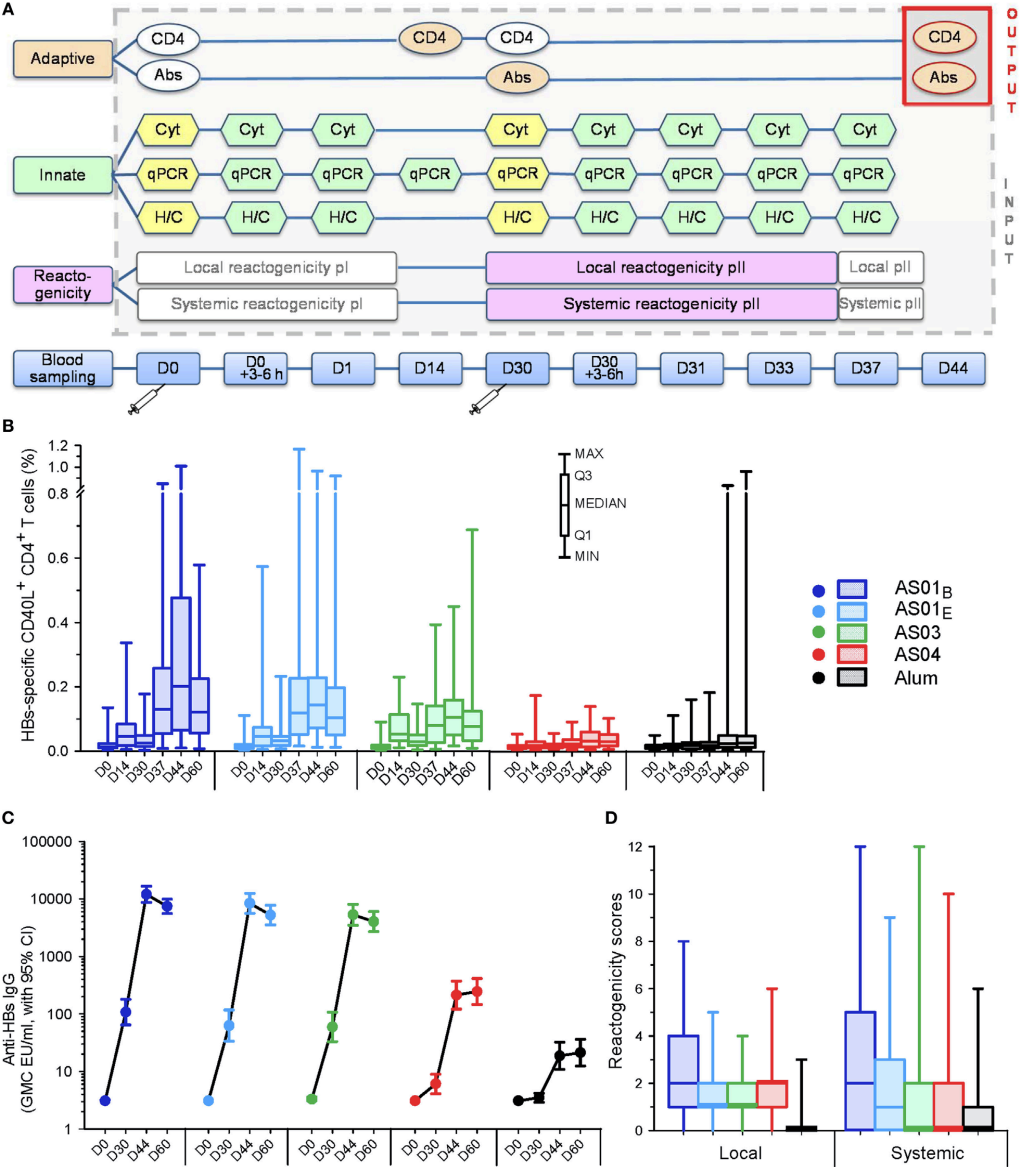
Multi-parametric analysis design and input parameters. **(A)** Study design schematic. Blood collection time-points for innate and adaptive immune response measurements and reactogenicity recordings are presented after the first and second vaccination (pI and pII, respectively) of hepatitis B surface antigen (HBsAg) adjuvanted with AS01_B_, AS01_E_, AS03_A_, AS04, or aluminum salt (“Alum”). Adaptive responses pII (“output”) expressed as frequencies of HBsAg-specific CD40L^+^ CD4^+^ T cells (CD4) or concentrations of HBs-specific antibodies (Abs) were modeled as a function of the innate immune responses pI or pII and reactogenicity pII (“input”). Innate parameters included cytokine/chemokine concentrations in serum (Cyt), gene expression assessed by qPCR in whole blood, and hematology and C-reactive protein measurements (H/C). Innate responses were expressed as fold-changes from baseline values (represented by the yellow shapes). White shapes represent values that were measured/recorded but not included in the multi-parametric analyses. Syringes indicate time-points of vaccination at days 0 and 30. Panels **(B–D)** represent the HBs-specific CD40L^+^ CD4^+^ T-cell responses and anti-HBs antibody responses (*N* = 286) through D60, and the local and systemic reactogenicity scores (*N* = 291) for the pooled time-points of the reporting period after the second vaccination, respectively. GMC, geometric mean concentration. CI, confidence interval.

Adaptive responses after the second injection (HBs-specific CD40L^+^ CD4^+^ T-cell frequencies or antibody concentrations at day 44) were modeled separately, as a function of the innate responses after the first (pI) or the second (pII) injection, and of reactogenicity pII (Figure 1A). The output parameters were selected as displaying the highest difference between the AS groups (34), and, for anti-HBs antibodies, serving as a proxy for protective immunity. Modeling was performed on participants of the ATP cohorts for whom data were available for each time-point and variable of immunogenicity and reactogenicity evaluations. Thus, each dataset in the model had the same size for the collective innate or adaptive responses and reactogenicity data (i.e., *N* = 256 or *N* = 84 for models including clinical laboratory/serum data or gene expression data, respectively; Table 1).

Solicited local AEs (pain, redness, swelling) and solicited systemic AEs [fatigue, fever (axillary temperature ≥ 37.5°C), headache, malaise, myalgia] as described previously (34) were represented in the model by the sum of either all individual local scores, or all individual systemic scores, by treatment group. These scores were derived from the maximum AE grading [based on the intensity grading described in Ref. (34)] reported by subject over all local or all systemic AEs. Of the 14-day safety follow-up period pII (from day 30 through day 43), only the first week (from day 30 through day 36) was considered in the modeling, since in the vast majority of subjects the solicited local and general AEs had resolved by day 6 post vaccination (34).

Principal component analysis was performed on the clinical laboratory/serum and qPCR datasets, with data for all variables and time-points expressed in FCs over pre-vaccination as described. Data were organized in a matrix with *n* rows (one per subject) and K*T columns of [VAR1_time1, VAR1_time2, …, VAR_K_timeT], where *K* = number of variables and *T* = number of post-vaccination time-points. The first three PCs (PC1, PC2, and PC3) of each dataset were included in the model.

#### Multi-Parametric Analyses

In the linear regression models, the adaptive response pII of each subject (A_pII,i_) was modeled as a function of: the regression coefficients β for the local and systemic reactogenicity scores pII by subject (LR_pII,i_ and SR_pII,i_), the individual innate variables pII (IN_pII,i_) as summarized by their first three PCs, and the treatment effect on the innate responses (per AS group relative to the Alum group), corrected for the error term (ε_i_). The Alum group was the primary comparator as displaying the lowest overall responses among groups and the β of its intercept (β_0_) was considered as the baseline. The following equation was used:

$$\begin{array}{l} {\text{A}}_{\text{pII,i}}={\text{β}}_{0}+{\text{β}}_{\text{LRpII}}\times {\text{LR}}_{\text{pII,i}}+{\text{β}}_{\text{SRpII}}\times {\text{SR}}_{\text{pII,i}}+{\text{β}}_{\text{PC}1[\text{INpII}]}\times \text{PC}1\left[{\text{IN}}_{\text{pII,i}}\right]+{\text{β}}_{\text{PC}1[\text{INpII}]}\\ \;\times \text{PC}2\left[{\text{IN}}_{\text{pII,i}}\right]+{\text{β}}_{\text{PC}3[\text{INpII}]}\times \text{PC}3\left[{\text{IN}}_{\text{pII,i}}\right]+{\text{β}}_{\text{AS}01\text{B}}\times {\text{IN}}_{\text{pII,AS}01\text{B},\text{i}}+{\text{β}}_{\text{AS}01\text{E}}\times {\text{IN}}_{\text{pII,AS}01\text{E,i}}\\ \;+{\text{β}}_{\text{AS}03}\times {\text{IN}}_{\text{pII,AS}03,\text{i}}+{\text{β}}_{\text{AS}04}\times {\text{IN}}_{\text{pII,AS}04,\text{i}}+{\text{ε}}_{\text{i}}. \end{array}$$

In a second model, the impact of the innate responses pI was evaluated by replacing the innate response PCs for the time-points pII by those for the time-points pI:

$$\begin{array}{l} {\text{A}}_{\text{pII,i}}={\text{β}}_{0}+{\text{β}}_{\text{LRpII}}\times {\text{LR}}_{\text{pII,i}}+{\text{β}}_{\text{SRpII}}\times {\text{SR}}_{\text{pII,i}}+{\text{β}}_{\text{PC}1[\text{INpI}]}\times \text{PC}1\left[{\text{IN}}_{\text{pI,i}}\right]+{\text{β}}_{\text{PC}2[\text{INpI}]}\\ \;\times \text{PC}2\left[{\text{IN}}_{\text{pI,i}}\right]+{\text{β}}_{\text{PC}3[\text{INpI}]}\times \text{PC}3\left[{\text{IN}}_{\text{pI,i}}\right]+{\text{β}}_{\text{AS}01\text{B}}\times {\text{IN}}_{\text{pI,AS}01\text{B,i}}+{\text{β}}_{\text{AS}01\text{E}}\times {\text{IN}}_{\text{pI,AS}01\text{E,i}}\\ \;+{\text{β}}_{\text{AS}03}\times {\text{IN}}_{\text{pI,AS}03,\text{i}}+{\text{β}}_{\text{AS}04}\times {\text{IN}}_{\text{pI,AS}04,\text{i}}+{\text{ε}}_{\text{i}}. \end{array}$$

In a third model, the impact of the adaptive response pI was evaluated by adjusting the first model for individual responses of HBs-specific CD40L^+^ CD4^+^ T-cell pI (CD4_pI,i_) and HBs-specific antibodies pI (AB_pI,i_) responses pI, as follows:

$$\begin{array}{l} {\text{A}}_{\text{pII},\text{i}}={\text{β}}_{0}+{\text{β}}_{\text{LRpII}}\times {\text{LR}}_{\text{pII,i}}+{\text{β}}_{\text{SRpII}}\times {\text{SR}}_{\text{pII,i}}+{\text{β}}_{\text{PC}1[\text{INpII}]}\times \text{PC}1\left[{\text{IN}}_{\text{pII,i}}\right]+{\text{β}}_{\text{PC}1[\text{INpII}]}\\ \;\times \text{PC}2\left[{\text{IN}}_{\text{pII,i}}\right]+{\text{β}}_{\text{PC}3[\text{INpII}]}\times \text{PC}3\left[{\text{IN}}_{\text{pII,i}}\right]+{\text{β}}_{\text{AS}01\text{B}}\times {\text{IN}}_{\text{pII,AS}01\text{B,i}}+{\text{β}}_{\text{AS}01\text{E}}\times {\text{IN}}_{\text{pII,AS}01\text{E,i}}\\ \;+{\text{β}}_{\text{AS}03}\times {\text{IN}}_{\text{pII,AS}03,\text{i}}+{\text{β}}_{\text{AS}04}\times {\text{IN}}_{\text{pII,AS}04,\text{i}}+{\text{β}}_{\text{CD}4,\text{pI}}\times {\text{CD}4}_{\text{pI,i}}+{\text{β}}_{\text{AB,pI}}\times {\text{AB}}_{\text{pI,i}}+{\text{ε}}_{\text{i}}. \end{array}$$

The resulting A_pII,i_ values were then included in a model to study the relationships between the parameters. The strength of a given association was described by an estimate of its effect size, variability (standard error; SE), and statistical significance, as summarized by the *p*-value. PCs with *p* < 0.05 were considered significantly associated with the adaptive responses.

## Results

To elucidate the role of early responses in vaccine immunogenicity, we modeled the adaptive responses after two injections as a function of both the innate response (measured after the first or second injection) and the reactogenicity scores reported after the second injection (Figure 1A). We used multi-parametric analyses to examine the strengths of the linear associations between these parameters, in terms of their estimated effect sizes, variability, and statistical significance, as determined for the AS groups relative to the Alum group (considered as the baseline). For the monitoring of the innate response, two separate analyses were performed on the per-protocol cohorts: one including clinical laboratory parameters and serum proteins (*N* = 291), and the second including gene expression data (*N* = 112; Table 1).

The adaptive responses were represented by either HBs-specific CD40L^+^ CD4^+^ T-cell or antibody responses measured 2 weeks after the second immunization (day 44), which were modeled separately since only limited associations between these responses were observed previously (34). The data reflected similar trends between adjuvant groups as reported previously for a larger cohort (34) (Figures 1B,C). The local and systemic reactogenicity scores reported after dose 2 also exhibited a comparable adjuvant ranking, but with greater similarity between the AS03 and AS04 groups (Figure 1D).

### AS Induce a Transient Increase in Levels of Innate Blood Parameters

**Figure 2:**
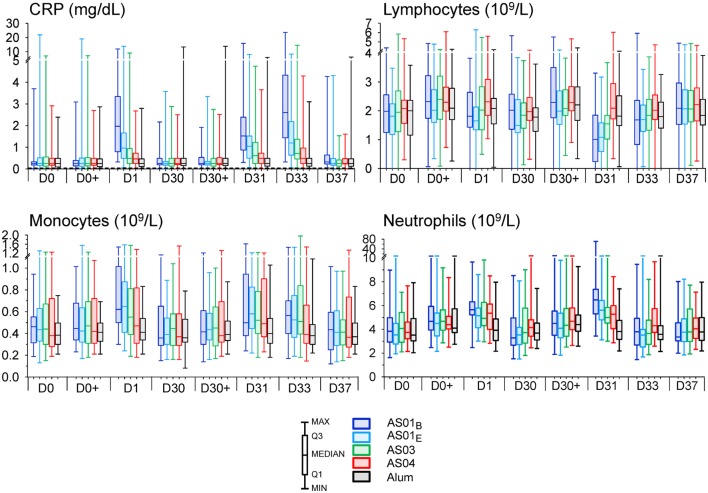
Innate hematology and C-reactive protein (CRP) responses. Normalized CRP concentrations (*N* = 59, 57, 59, 61, and 55) and normalized lymphocyte, monocyte, and neutrophil counts (*N* = 38, 40, 45, 39, and 41) in the AS01B, AS01E, AS03, AS04 and Alum groups, respectively, of the per-protocol cohort for innate immunogenicity are represented in box-whisker plots with medians, interquartile ranges, minima and maxima indicated. D, day.

**Figure 3:**
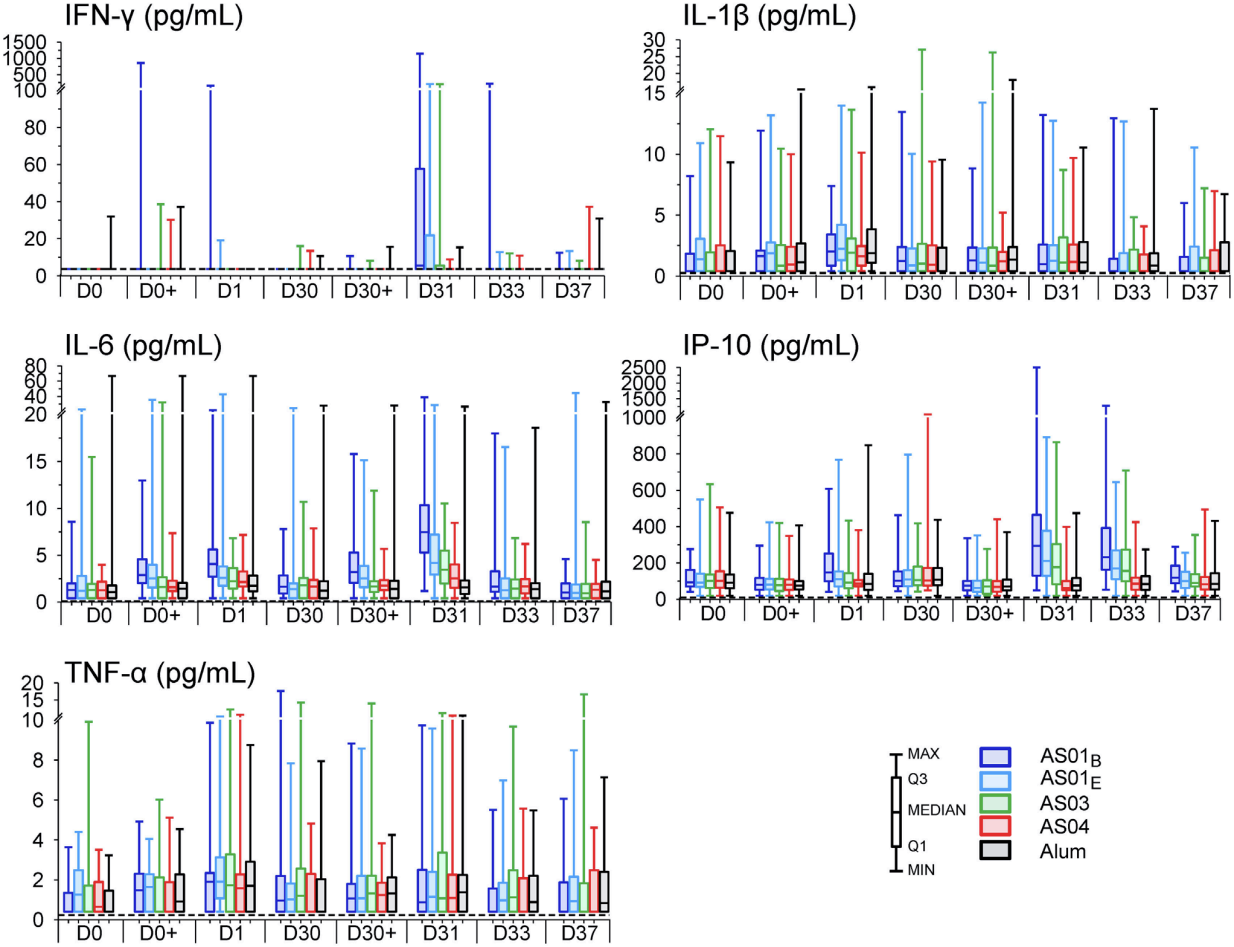
Innate cytokine responses. Interferon (IFN)-γ, interleukin (IL)-1β, IL-6, induced protein (IP)-10, and tumor necrosis factor (TNF)-α concentrations for the AS01_B_, AS01_E_, AS03, AS04 or Alum groups of the per-protocol cohort for innate immunogenicity (N = 59, 57, 59, 61 and 55, respectively) are represented in box-whisker plots with medians, interquartile ranges, minima and maxima indicated. The dotted lines indicate the assay cutoffs.

To highlight the innate parameters most impacted by the adjuvanted vaccines, the responses were first expressed as median FCs from the two pre-vaccination time-points (days 0 and 30), and represented as heat maps [considering biological significance at a FC of ≥|1.5| (Figures S1A,B in Supplementary Material)]. Absolute values of a selection of these parameters are presented in Figures 2 and 3.

Evaluation of the clinical laboratory data revealed CRP responses at 1 and 3 days post vaccination (Figure S1A in Supplementary Material). Indeed, median CRP levels were increased in the AS01 and AS03 groups at day 1, and in each AS group at days 31 and 33 (10-, 5-, 3- and 2-fold for AS01_B_, AS01_E_, AS03 and AS04, respectively at day 33). Changes in blood cell counts were detected mainly at day 1 post each vaccination (Figure 2). All parameters returned to baseline within 1 week post vaccination (Figure S1A in Supplementary Material; Figure 2). Among the myeloid lineage, the increase in neutrophil counts after the second dose was the most prominent signature. While this increase was, in fold-changes, only seen at day 31 in the AS01_B_ and AS01_E_ groups (1.8- and 1.5-fold, respectively), evaluation of the interquartile ranges of the absolute neutrophils and monocytes counts revealed a trend for increased responses in all AS groups at both day 1 and day 31. The monocyte counts tended to remain slightly elevated through day 33 in the AS01 and AS03 groups and returned to baseline at day 37. Likely associated with this increase in myeloid cells at day 31 was the concurrent transient decrease in the relative lymphocyte fractions, which was, in fold-changes, observed in the AS01_B_ group (−1.6-fold), and, in absolute counts, also in the AS01_E_ and AS03 groups. No clear CRP or hematology responses were observed in the Alum group.

All formulations triggered transient cytokine responses (Figure S1B in Supplementary Material). After the first injection, at 3–6 h, only IL-6 was detected in the AS01_B_ and AS01_E_ groups (3- and 1.6-fold, respectively). At day 1, most of the pro-inflammatory markers measured (listed in Table S1 in Supplementary Material) were increased in at least one group, of which the IL-6 and TNF-α levels were increased in all groups (1.6- to 4-fold, and 1.7- to 2-fold, respectively; Figure S1B in Supplementary Material). The IL-6 levels in the AS01_B_ group surpassed those in the Alum group at both time points (*p* = 0.001). Of note, median IL-5, IL-1β, and IL-10 responses were ambiguous, since the individual concentrations often approached the assay cutoffs. After the second injection, IL-6 levels were increased at 3–6 h on day 30 in the AS01 groups (1.9-fold) and at day 31 in each AS group (1.7- to 4-fold), and had returned to baseline at day 33. At 3–6 h on day 30 and at day 31, IL-6 levels in both AS01 groups exceeded those in the Alum group (*p* = 0.001).

Changes in IP-10 and IFN-γ levels were only observed after the second injection. Of these responses, IP-10 levels were only increased in the AS01 groups at days 31 and 33 (1.6- to 3-fold) and were at both of these time-points significantly different from the decreased levels in the Alum group (i.e., −1.6 and −1.47, respectively; *p* ≤ 0.002). These responses had subsided to baseline at day 37. IFN-γ was only increased in the AS01_B_ group at day 31 (1.5-fold). MCP-1 levels were decreased in all groups, and predominantly at day 33.

Evaluation of the absolute cytokine concentrations revealed that the variability was relatively high across subjects, groups, and parameters (Figure 3). Interestingly, discrete IFN-γ responses were observed in some individuals of each group. In particular, in 14% of the participants who received AS01, IFN-γ levels were already detectable at day 1 (Figure S2A in Supplementary Material).

### Early Changes in CRP, IL-6, IFN-γ, and IP-10 Levels Are Associated with the Magnitude of the Adaptive Response

**Figure 4:**
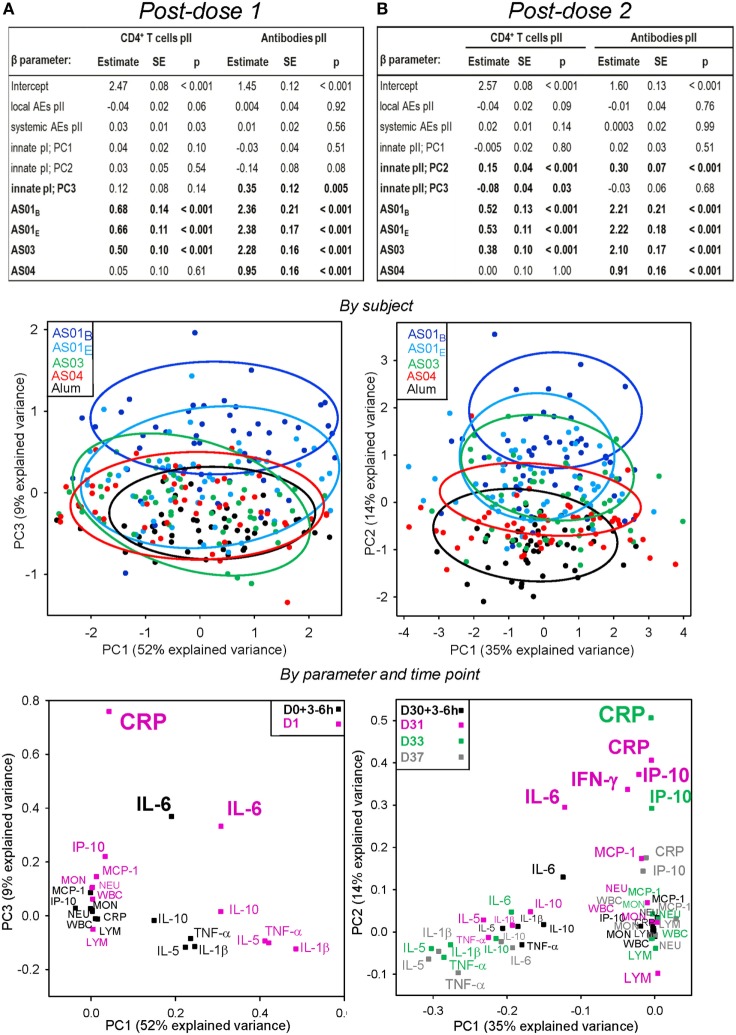
Associations between adaptive responses, and both the innate responses of hematology, C-reactive protein (CRP) and serum protein variables, and reactogenicity. Participants received HB surface antigen adjuvanted with AS01_B_, AS01_E_, AS03, AS04, or aluminum salt (Alum; *N* = 53, 53, 52, 51, and 47, respectively) at days 0 and 30. Data obtained after the first (pI) or the second injection (pII) are shown in panels **(A,B)**, respectively. Upper panels: multi-parametric analyses of adaptive responses were performed for each coefficient (β) of the listed model input parameters, in terms of the estimate of the effect size, standard error (SE), and *p*-value (*p*). Input parameters included local and systemic reactogenicity scores calculated from the solicited adverse events (AEs) pII, and the innate responses pI and pII. Intercept, β for Alum group (β_0_). Adjuvant systems (AS) groups were compared with the Alum group (considered as baseline). HBs-specific CD4^+^ T-cell and antibody responses were measured at day 44. Principal components (PCs) that were significantly associated (*p* < 0.05) with the adaptive response are indicated by bold font. Middle panels: PC analysis of the innate response dataset was performed by subject and treatment group and visualized in bivariate plots. The variance explained by the first three PCs was 73% after the first injection and 58% after the second injection. Each dot represents the expression profile of an individual subject. Arbitrary aggregation of the subjects into treatment groups is visualized by the colored ellipses, according to the color coding presented in the left-hand corners of the plots. The PC1 accounted for 52 and 35% of the variance after the first and second dose, respectively, and is plotted against the PCs showing the strongest association with the adaptive response in the table in the upper panels, i.e., the PC3 after the first dose and the PC2 after the second dose. Lower panels: as for the middle panels, but with PCs representing the variables at the post-vaccination time-point indicated by the color coding in the upper corners of the PC plots. An overview of PC plots for each PC1, PC2, PC3 combination by variable/time-point is presented in Figure S3 in Supplementary Material. LYM, lymphocytes; MON, monocytes; NEU, neutrophils; WBC, white blood cells.

We next tested our hypothesis that adaptive and early innate responses after vaccination are associated. In order to perform association analyses, the innate parameters were summarized by PC analysis and the first three PCs were used in the multi-parametric model. After the first vaccine dose, and following adjustment for treatment effect, no association between the PCs representing the innate responses, and the CD4^+^ T-cell response at day 44, could be found (Figure 4A). As expected, significant associations were seen between the CD4^+^ T-cell responses and the AS01 and AS03 treatments, as well as between the antibody responses and all four AS treatments (*p* < 0.001; *upper panel*). Weaker associations were seen between the CD4^+^ T-cell responses and systemic reactogenicity (*p* = 0.03), and between the antibody responses and the PC3 of the innate response (*p* = 0.005). Visualization of this PC3 in a PC1, PC3 plot allowed grouping of the individual subjects with overall similar expression profiles (*middle panel*). Consistent with the cytokine expression profiles of the AS after the first dose (see Figure S1B in Supplementary Material and Figure 3), the patterns of all AS groups were largely overlapping, with only the AS01_B_ and Alum groups exhibiting a clear separation on the PC3. To identify the relative contributions of the individual variables to the statistically significant association observed for the PC3, as well as to represent the association between different parameters, we visualized the loadings of the analyte–time-point combinations in a second PC1, PC3 plot (Figure 4A, *lower panel*). IL-6 at 3–6 h and day 1 and CRP at day 1 exhibited the strongest separation on the PC3 axis and were consequently most strongly associated with antibody response. In line with the data in Figures S1A,B in Supplementary Material, these parameters were mostly activated by AS01_B_.

When the innate responses after the second injection rather than those after the first injection were included in the model (Figure 4B), stronger associations of CD4^+^ T-cell and antibody responses with the PC2 (*p* < 0.001) and, to a lesser extent, the PC3 (*p* = 0.03; CD4^+^ T cells only) were seen (*upper panel*). No associations with reactogenicity were observed. A PC1, PC2 plot showed that the subjects clustered largely separately from each other by group, with less overlap than was observed after the first dose (*middle panel*). Loading analyses by analyte and time-point along the relevant PCs revealed that for the PC2, the association engaged more variables than after the first dose (*lower panel*). The largest separations on the PC2 were observed for IL-6 and IFN-γ at day 31, and CRP and IP-10 at days 31 and 33. A PC1, PC3 plot revealed that the weak association of the CD4^+^ T-cell response with the PC3 was mostly determined by a cluster formed by TNF-α, IL-5, IL-1β, and IL-10 at day 31 (Figure S3 in Supplementary Material). Thus, key innate markers associated with the magnitude of adaptive responses were, after the first dose, CRP (at day 1) and IL-6 (at 3–6 h or day 1), and after the second dose, CRP and IP-10 (at days 31 and 33) and IFN-γ and IL-6 (at day 31). Of note, other important markers, such as neutrophil or monocyte counts (which were both increased post vaccination), were not associated with the adaptive response.

To evaluate the impact of the adaptive response to the first vaccine dose on the adaptive response to the second vaccine dose, day 14 CD4^+^ T-cell responses and day 30 antibody responses were added to the model (Table S2 in Supplementary Material). The levels of the adaptive response after the first dose were associated with the levels of both antibody and CD4^+^ T-cell responses after the second dose (*p* ≤ 0.04; |β| range: 0.1–0.4). However, associations with the innate parameters after two doses were no longer observed, suggesting that the influence of the adaptive response after one vaccine dose on the day 44 adaptive response was stronger than that of the innate response.

### Gene Expression Analysis Supports the Association of Early IFN Pathways and Adaptive Response

To further explore our hypothesis to the gene expression level, we quantified the expression of 14 genes encoding major cytokines or transcription factors implicated in inflammation, cell proliferation, and the IFN pathway (listed in Table S1 in Supplementary Material), by whole blood qPCR for a subset of subjects (*N* = 112). This selection was based on published literature describing either innate responses to different AS-containing vaccines and other vaccines in humans (31, 35, 37, 38) and animal models (25–27), or early IFN-related responses to AS01-adjuvanted candidate vaccines in humans (32, 35, 39) and animals (40).

**Figure 5:**
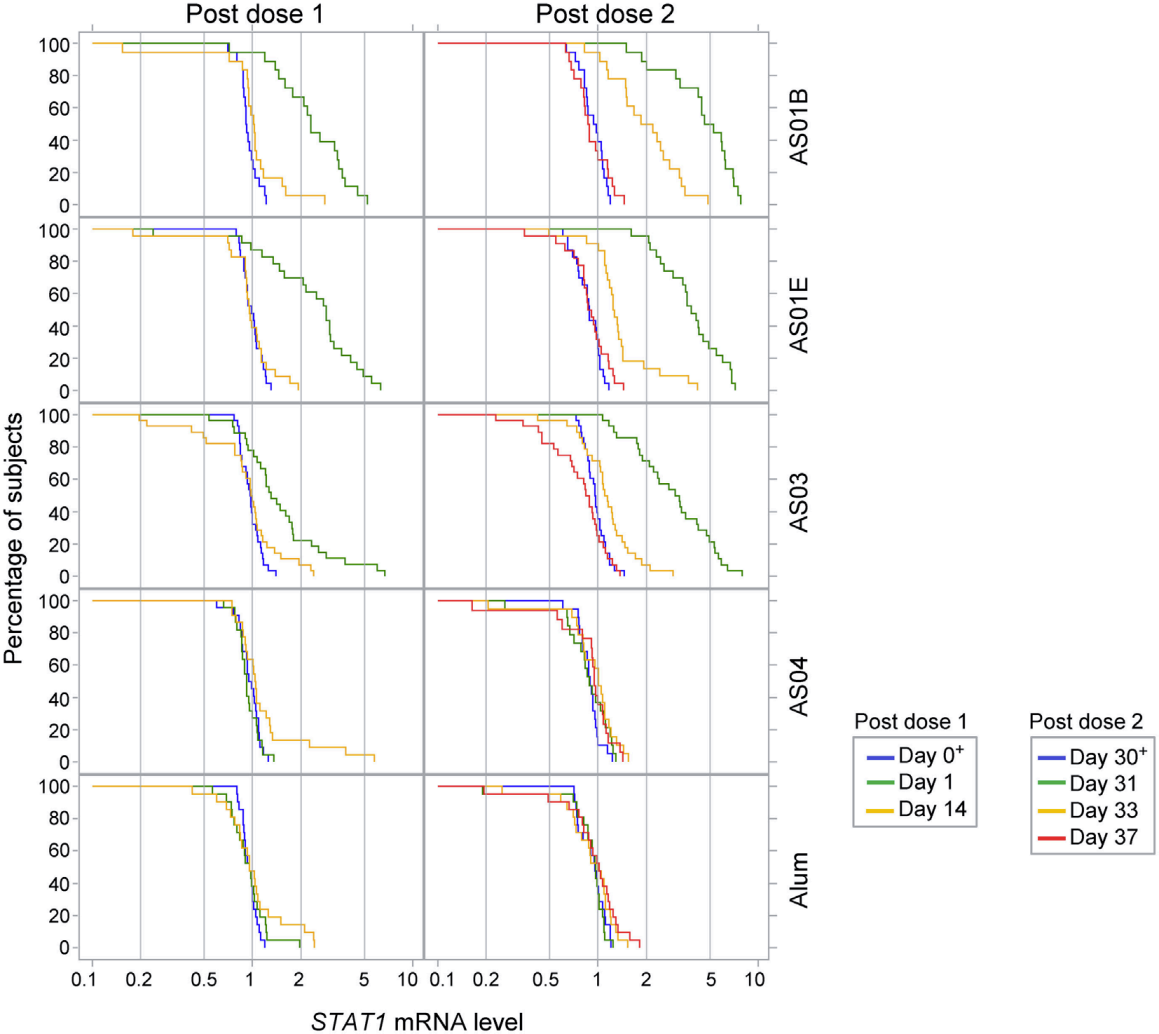
STAT1 expression. Reverse cumulative distribution curve of the *STAT1* mRNA responses (in FC over day 0 or day 30 levels; *N* = 18, 23, 28, 22 and 21 in the AS01B, AS01E, AS03, AS04 and Alum groups, respectively) in whole blood after the first and second vaccine dose (left and right parts of the figure, respectively) are presented for the time points up to day 37.

No changes in gene expression patterns were seen in the Alum and AS04 groups after either dose (Figure S1C in Supplementary Material). Median responses in the other groups were only seen at day 1 (AS01 groups), day 31 (all three groups), and day 33 (AS01_B_ group only), with comparable signatures between the AS01_B_ and AS01_E_ groups. After the first dose, at day 1, only *STAT1* was slightly upregulated in the AS01_B_ and AS01_E_ groups (2- and 3-fold, respectively). Of note, increased *STAT1* mRNA levels after the first vaccine dose were also seen in a minority of individuals in the AS03 group (Figure 5). After the second dose, at day 31, upregulation of the IFN-inducible genes *STAT1, IRF1, MX1*, and *CXCL10* was observed in the AS01 groups (4- or 5-, 2.6- or 3-, 2- or 3-, and 3- or 6-fold, respectively; Figure S1C in Supplementary Material), consistent with the concurrent detection of IFN-γ (AS01_B_ group) and IP-10 (both AS01 groups) proteins in serum (see Figure S1B in Supplementary Material; Figure 3). Yet, no change in the levels of *IFNG* mRNA could be detected at any time point (Figure S2B in Supplementary Material). At day 33, only the *STAT1* and *MX1* upregulation in the AS01_B_ group persisted (Figure S1C in Supplementary Material). Interestingly, a slight downregulation of *NFATc2*, a gene that is upregulated in T lymphocytes, was detected at day 31. This coincided with the observed decrease in lymphocyte counts at the same time point (see Figure S1A in Supplementary Material; Figure 2) and potentially reflected the recruitment of antigen-specific T cells (and bystander cells) to the dLN. In the AS03 group, only the median *MX1* and *STAT1* mRNA levels were increased, at day 31 (2- and 3-fold, respectively). In sum, the AS01- or AS03-adjuvanted vaccines shared the same INF-related signature at day 31 (although more and stronger signals were detected with AS01), while the AS04- or Alum-adjuvanted vaccines had no apparent effect on the whole-blood gene expression.

**Figure 6:**
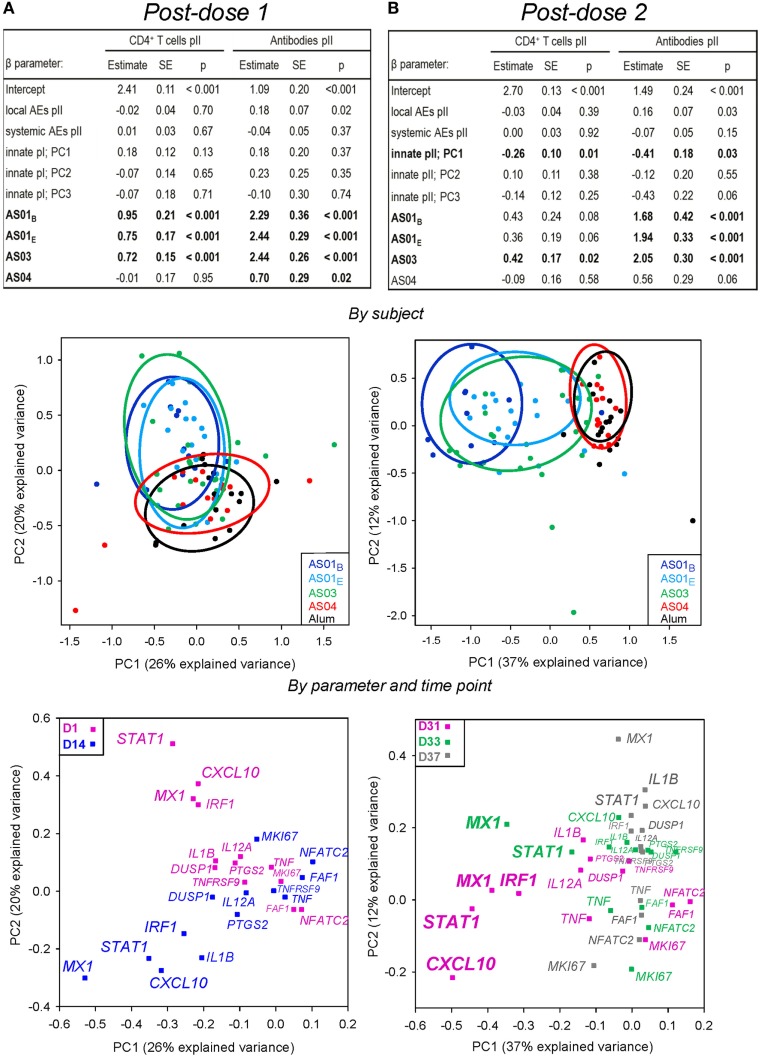
Associations between the adaptive responses, and both the innate mRNA responses and reactogenicity. As for Figure 4, for gene expression in the AS01_B_, AS01_E_, AS03, AS04, or Alum groups (*N* = 11, 18, 23, 15, and 17, respectively). Results show data obtained after the first **(A)** or second **(B)** injection. Upper panels: multi-parametric analysis of the adaptive response to vaccination was performed for each coefficient (β) of the listed model input parameters, in terms of the estimate of the effect size, standard error (SE), and *p*-value (*p*). Input parameters included the local and systemic reactogenicity scores calculated from the respective solicited adverse events (AEs) post-dose 2 and innate responses after each dose. Intercept, β for Alum group (β_0_). Adjuvant systems (AS) groups were compared with the Alum group (considered as baseline). HBs-specific CD4^+^ T-cell and antibody responses were measured at 2 weeks post-dose 2. PCs that were significantly associated (*p* < 0.05) with adaptive responses are indicated by bold font. Middle panels: principal component (PC) analysis of post-vaccination innate responses was performed by subject. The variance explained by the first three PCs was 55% post-dose 1 and 59% post-dose 2. Each dot represents the expression profile of an individual subject. Aggregation of the subjects into treatment groups is arbitrarily visualized by the colored ellipses, according to the color coding shown in the left-hand corners of the plots. The PC1 accounted for 26 and 37% of the variance after the first and second dose, respectively, and is plotted against the PC showing the strongest association with adaptive responses in the tables shown in the upper panels, i.e., the PC2 after each dose. Lower panels: as for the middle panels, but with the PCs representing the variables at the post-vaccination time-points indicated by the color coding in the upper corners of the plots. An overview of the PC plots by variable/time-point for each PC1, PC2, PC3 combination is presented in Figure S4 in Supplementary Material.

Multi-parametric analyses of the adaptive responses for a subset of subjects (*N* = 84) revealed only a weak association with the PC1 of the innate responses post dose 2 (*p* = 0.01 or *p* = 0.03; Figures 6A,B; *upper panels*). As expected, the PC1, PC2 plots after each dose showed that the AS01 and AS03 treatments were separated from the AS04 and Alum treatments (*middle panels*). As shown in the PC1, PC2 (*lower panel*) and PC1, PC3 (Figure S4 in Supplementary Material) plots by variable, the association with the PC1 of the innate responses was predominantly governed by *STAT1, IRF1, MX1*, and *CXCL10* at day 31, and *MX1* at day 33. Thus, upregulation of IFN-inducible genes post dose 2, as induced by AS01 and AS03, was associated with higher adaptive responses.

When the model was adjusted for adaptive responses post dose 1, the latter responses but not the innate responses were significantly associated with the adaptive responses post dose 2 (Table S2 in Supplementary Material), confirming the observations made for the clinical laboratory/serum dataset.

## Discussion

Previously, we described the reactogenicity and adaptive responses induced by HBsAg adjuvanted with AS01_B_, AS01_E_, AS03, AS04, or Alum, in close to 600 young, HBV-naïve adults (34). As part of this study, we also characterized the innate responses in blood collected from 291 of these participants and used these data in multi-parametric models of adaptive responses as described here. We found that the vaccines provoked transient responses which started at 3–6 or 24 h after vaccination. These responses comprised inflammatory markers (for all AS), neutrophils (for AS01), and mRNAs encoding cytokines implicated in the innate response (for AS01 and AS03). Yet, no unique protein, cellular, or gene expression signature was identified for one particular vaccine. Furthermore, adaptive responses post dose 2 were found to be associated with, in order of decreasing strength, adaptive responses post dose 1, then innate responses post dose 2, and then innate responses post dose 1, while reactogenicity was not identified as a significant predictor of adaptive responses. The association between innate and adaptive responses after two doses was largely driven by increased levels of CRP and IL-6 promoted by all AS, and of parameters of the IFN-signaling pathway promoted by AS01 or AS03. Importantly, the multi-parametric model proved to be able to specifically identify those innate parameters that were associated with the adaptive response. This was exemplified by the observation that for some innate parameters (e.g., neutrophil counts), the levels were markedly changed after the first and/or second vaccination, while the modeling showed associations with neither the CD4^+^ T-cell response nor the antibody response.

The adjuvant ranking for innate responses was overall similar to that observed previously for adaptive responses (34). The superior adjuvanticity of AS over Alum is overall consistent with mice data showing comparable differences between these adjuvants in innate cellular and cytokine responses in dLNs (25–27), and similar observations were made for other adjuvants (6). For AS04, the difference with AS01 may be explained by its lack of QS-21, although this comparison is hampered by the difference in the formulations of these AS. The presence of MPL in AS04 may explain its difference with Alum, but this comparison is confounded by the different aluminum salts these adjuvants contain. Among the AS01-adjuvanted vaccines, the magnitude of the innate response was overall higher with AS01_B_, and although this did not translate into significantly different adaptive responses, it was consistent with the lower inter-subject variability of the adaptive responses observed in the AS01_B_ group (34). This suggests that a more potent innate response can contribute to generate a more robust antigen-specific response, which is particularly relevant for individuals with a tendency to respond less efficiently to vaccines. For example, in evaluations of an varicella zoster virus glycoprotein E vaccine in older adults, who are anticipated to be less responsive to vaccines in general, the antibody and CD4^+^ T-cell responses were significantly higher for the AS01_B_-adjuvanted vaccine than for the AS01_E_-adjuvanted vaccine (41). This is consistent with a recent publication of Nakaya et al. showing that the impaired IFN-related genes signature following influenza vaccination in the elderly population in that study was associated with the decreased antibody response (42). This was, however, not seen in the responses induced by a tuberculosis vaccine (M72/AS01) in healthy adults (43), suggesting that this effect might be dependent on the immune status of individuals and the specific combination of antigen and adjuvant. Overall, our data suggest that for the current antigen and naive adult population, the potency of an AS in inducing systemic innate responses was positively correlated with the magnitude of adaptive responses.

Production of both IL-6, a cytokine promoting T helper (T_H_) cell stimulation, and CRP were features shared by the AS. This aligns with non-clinical data showing IL-6 responses in the mouse dLN, muscle and serum and/or in human primary cell cultures (for MPL, QS-21, or AS03), and CRP responses in rabbits (for AS01 and AS03) (25–28, 44). The time-courses for the sequential peaks of IL-6 (3–6 h) and CRP (24 h) were reminiscent of those found for a non-adjuvanted bacterial vaccine in humans (45). This sequence is also consistent with CRP production in the liver being, at least in part, under transcriptional control of the IL-6 pathway (46, 47). A hallmark of the response to the vaccines containing AS01 and AS03 (but not AS04 or Alum) was the upregulation of the IFN pathway, manifested by changes in the expression of IFN-related genes and increases in serum IFN-γ and IP-10 levels. IP-10 may be downstream of IFN signals, as it is produced by several cell types in response to IFN-γ or IFN-α (48, 49). Of interest, IFN-related protein and gene expression was also observed in the predominantly H1N1-primed recipients of AS03-adjuvanted H1N1 influenza vaccine (38). However, in the naïve participants of the present study, the IFN-γ response induced by the HBsAg/AS03 vaccine was less prominent and mainly seen after the second dose. Interestingly, in mouse models, AS01 has been shown to directly drive early IFN-γ production by both NK cells and CD8^+^ T cells, resulting from a synergistic effect of MPL and QS-21 on macrophages in the dLN, which eventually led to the synergistic enhancement of polyfunctional CD4^+^ T-cell responses (11, 40, 50). Furthermore, IFN-γ derived from innate immune cells enhances protective anti-parasitic Th1 responses (51), and recent data suggested a link between the detection of early IFN signatures in blood from RTS,S/AS01 vaccinees and their subsequent protection from malaria (52). These mechanistic clues to the mode of action of AS01 may explain why both AS01_B_ and AS01_E_ ranked highest among the AS with respect to IFN-related gene expression and IL-6 and CRP levels, and why they were eventually shown to be most strongly associated with adaptive responses.

**Figure 7:**
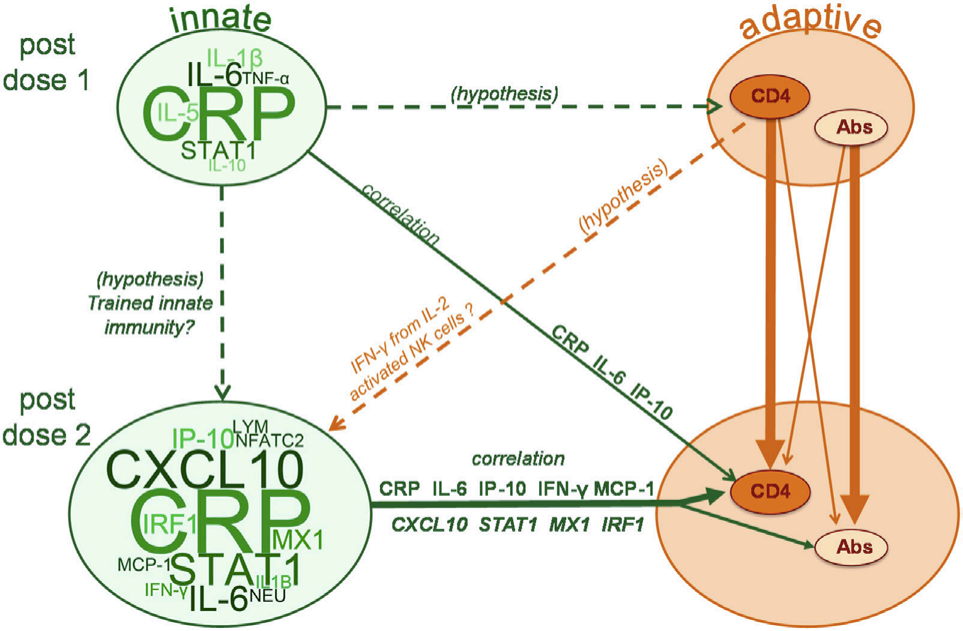
Model schematic of the interplay between innate and adaptive responses. Word clouds represent changes in levels of serum proteins, hematology parameters or mRNA levels, with cloud and font sizes proportional to the response levels presented for these variables in Figure S1. Solid arrows indicate significant associations observed in the multi-parametric models of adaptive responses to vaccination [i.e., responses of HBs-specific CD4_+_ T cells [CD4] or antibodies (Abs)], with line thickness proportional to the strength of the associations. Analytes presented on the solid arrows reflect those for which the strongest separation and highest loading was observed on the principal component found to be relevant for the association with adaptive responses. Dashed arrows indicate hypothetical associations that are discussed in the manuscript but not evaluated by the multi-parametric analyses. LYM, lymphocytes; NEU, neutrophils.

One of the aims of this study was to identify potential interrelationships between innate and adaptive responses. IFN-γ specifically is known to be critical for both adaptive and innate immunity. In addition to antigen-specific T cells, IFN-γ is secreted by innate lymphoid cells, such as innate-type T cells or NK cells, among others [reviewed in Ref. (53)], that can be involved in the onset of the immune response as well as in protection mechanisms. In our study, IFN-γ signaling pathways were found to be increased after the second dose vs after the first dose. Similar trends of increased serum IFN-γ after repeated administration of AS-adjuvanted vaccines were observed in clinical trials evaluating either HBsAg/AS01 (32), or other antigens (M72, RTS,S) combined with either AS02 (an emulsion containing MPL and QS21) or AS01 (35, 54). Moreover, peripheral IFNγ-producing NK cells were observed after M72/AS01 and RTS,S/AS01 vaccination in humans (55, 56). Taken together, the current observations with respect to IFN-associated responses may be explained by two, not mutually exclusive hypotheses, involving either “trained immunity” or a CD4^+^ T-cell regulated mechanism (Figure 7). Trained immunity, involving epigenetic imprinting of the IFN-γ loci in NK cells following activation, could drive higher IFN-γ production by memory NK cells upon re-challenge (2, 3, 5, 57). Alternatively or in addition, stimulation of IFN-γ secretion by NK cells or monocytes could be mediated by IL-2 produced by vaccine-induced effector memory T cells (55, 58–60). Since in our study, only AS01 and AS03 provoked IFN-associated responses, their efficiency in inducing adaptive responses may, thus, possibly be linked to their capacity to trigger IFN-signaling.

While we did not evaluate a putative association between inflammatory markers and reactogenicity, such association may plausibly exist, given the parallel trends in innate responses and the prevalence of reactogenicity events [i.e., both were higher with the formulations with AS vs Alum, and (for systemic reactogenicity) both were increased after the second dose of AS01_B_-adjuvanted vaccine (34)]. Yet, reports of immunological correlates of reactogenicity in humans are scarce and often conflicting. For instance, in recipients of non-adjuvanted influenza vaccine, local or systemic reactogenicity events corresponded with post-vaccination levels of MIF and/or TNF-α, but not of IL-6, IL-8, or IL-1β (61), and CRP increases seen in other clinical vaccine trials were also not clearly associated with reactogenicity (62–64). Furthermore, reports of severe AEs in recipients of AS03-adjuvanted A(H1N1)pdm09 influenza vaccine did not correlate with blood TNF, IL-6, IFN-γ, or CRP levels, nor, albeit ambiguous, with IP-10 levels (38). In non-clinical studies, however, the data suggested correlations between systemic IL-6 responses and increased body temperature (65), and between upregulation of IFN-inducible and innate phase genes and several reactogenicity parameters (66). Still, animal models do not faithfully mimic human responses to inflammation, as evidenced by the limited correlations between the human endotoxemia model and mouse endotoxin model (a common proxy for inflammation in humans) (67). Nonetheless, if innate immunity and reactogenicity are linked, reduction of reactogenicity while preserving immunogenicity poses an interesting opportunity for vaccine development. For AS01, this was addressed by the dose reduction from AS01_B_ to AS01_E_ for a candidate tuberculosis vaccine, which generally decreased reactogenicity without affecting adaptive response magnitudes (43). Importantly, the acceptable safety profiles described for the current AS01-, AS03-, or AS04-adjuvanted HBsAg vaccines [as described in Ref. (32, 34, 68)] were supported by our observation that the inflammatory responses typically returned to baseline within 1 or 3 days. Still, given the variability of the human population and limited number of markers assessed, the inflammatory marker signatures obtained in the current young, naive adult population cannot be extended to other populations, nor serve to predict risks of rare safety signals.

Previous preclinical assessment has shown that the effect of AS is local, as the antigen needs to be co-localized with the adjuvant at the site of injection, presumably engaging the same dLN (25–27). As we only probed blood in the current study, the systemic data may not fully reflect the direct events occurring in the muscle at the site of injection and the dLN. For example, in AS04-treated mice, the cytokine concentrations measured locally were ~10-fold higher than those in peripheral blood (25). Nonetheless, the changes in *STAT1, CXCL10*, and *IRF1* mRNA and concurrent IFN-γ and IP-10 responses suggested that the IFN-γ canonical pathway was activated in circulating cells. This is supported by the fact that monocytes and lymphocytes can produce IP-10 in response to IFN-γ responses. Since *STAT1* is transcriptionally activated by IFN-γ as well as by IFN-α/β (which were not measured) a role of type-1 IFNs cannot be excluded. Cytokine consumption or dilution effects from using whole blood instead of sorted cells may also have been confounding factors. Overall, because no changes in *INFG* mRNA were detected, the systemic IFN-γ responses may indicate local production in the injected muscle or dLN, as supported by the IP-10 protein and *CXCL10* mRNA responses.

The PC modeling revealed overlapping innate immune profiles for a portion of the participants in the AS01, AS03, and AS04 groups. Furthermore, AS01 and AS03 were shown to display similar innate marker profiles, although the responses they induced were of different magnitudes. Such resemblance was not obvious from previous evaluations of these adjuvants in preclinical models, in which the set of markers partly overlapped the selection assessed in the current study (26–28). Comparative analyses of several adjuvants in mice revealed that, while the adjuvants shared a core inflammatory signature, they also displayed distinctly different specific signatures (6, 69). Yet, the relevance of such adjuvant-specific signatures obtained in inbred mouse models for human vaccine studies remains unclear, necessitating further comparative evaluations in a clinical context. Furthermore, it is likely that the current observations are at least partially a consequence of our marker selection. Indeed, the input data of the model assessing gene expression were generated by qPCR, a method chosen for its robustness, and included a limited selection of genes, to gain sufficient power in the modeling. As a consequence and potential limitation, the number of genes was lower as compared to that in assays evaluating genome-wide expression. Hence, as a next step, we are currently in the process of generating microarray data for this study, focusing on both the group-averages and the individual participants’ gene expressions. These data may serve to (1) confirm the current observations, (2) uncover specific differences in the modes of action of AS01, AS03, and AS04 underlying the differential impacts these AS were shown to have on the adaptive response. Similarly, more work is needed to better define any potential differences between these adjuvants in the quality of the induced antigen-specific responses, including the breadth, avidity, polyfunctionality, memory phenotype, or persistence, which were previously shown to be altered by other adjuvants (32, 70–73). It will also be of interest to define such differences as a function of the physicochemical properties of these adjuvants. The kinetics of the innate response induced by the different AS described here has been studied mainly in mice (25–27), rabbits (28), and sheep (74). These animal data showed that all AS trigger a transient innate response regardless of their composition. In AS04, MPL is adsorbed on Alum, but the kinetics of the innate immune response induced by MPL is not significantly impacted by the depot effect of Alum (26). The difference in the compositions of AS01 (liposome-based) and AS03 (oil-in-water emulsion) is reflected in discrepancies of their respective response. In rabbits, the CRP levels induced by AS03 declined at a slower rate as compared to those induced by AS01 (28), which may be related to the greater retention of the oil-in-water emulsion at the injection site (29). By contrast, AS01 rapidly drained to the local lymph node (26), and 3 days after injection no signs of inflammation were detected at the injection sites of AS01-treated rabbits (75). While this may be inconsistent with the similar kinetics of the AS-adjuvanted vaccines observed here, it could also be a function of the selected time points, or of biological differences between animals and humans.

Because the study was designed to allow a head-to-head comparison of the innate responses to the adjuvanted vaccines, neither placebo controls nor adjuvant- or antigen-only groups were included in the study design. Therefore, the data cannot serve to ascertain the impacts of the needle insertion or the HBsAg by itself. *Engerix-B* was used as an adequate benchmark because it is the current standard of prevention against hepatitis B viral infection. In addition, the use of Alum as a benchmark adjuvant is relevant because Alum is used in many vaccines and has a well-established safety profile. *Engerix-B* had little impact on the innate markers studied here, in agreement with murine data (27), suggesting that the antigen itself would have limited impact on the innate response directly. Collectively, this suggests that the innate response induced by the AS-containing HBsAg vaccines were directly attributable to these adjuvants.

## Conclusion

A feature shared by AS01, AS03, and AS04 is that the innate immune responses promoted by these adjuvants in humans led to increased adaptive responses to the co-administered antigen, confirming the mechanism of action of these adjuvants investigated in animal models. Despite the distinctly different compositions of these adjuvants, the innate immune responses activated by AS01 and AS03 converged toward a common pathway, the IFN pathway, which was associated with enhanced adaptive responses.

## Trademark Statements

FENDrix and Engerix-B are trademarks of the GSK group of companies.

## Ethics Statement

The protocol was approved by all institutional Ethics Committees and conducted in accordance with the Helsinki Declaration and Good Clinical Practice guidelines.

## Author Contributions

WB, AD, SD, NG, GL-R, AM, and RM participated in the conception, planning, and/or design of the study. SD, VB, RB, and MJ participated in the data generation. WB, VB, FC, AD, NG, MJ, GL-R, AM, RB, and RM performed or supervised the analysis of data and interpreted the results. AC and LF provided with statistical expertise for the modeling and/or analysis and interpretation of the results. GL-R was the principal investigator of CEVAC and FC coordinated CEVAC’s participation. WB led the development of the outline. All authors participated in the development of this manuscript. All authors had full access to the data, gave final approval before submission, and agreed to be accountable for all aspects of the work. The corresponding author was responsible for submission of the publication.

## Conflict of Interest Statement

AC, AD, LF, MJ, RB, RM, VB, and WB are employees of the GSK group of companies. AD, RB, RM, VB, and WB hold shares in the GSK group of companies as part of their employee remuneration. AD and RM have a patent on AS01 (fractional dose) pending. AM’s institution received payment from the GSK group of companies for the performance of the clinical trial as well as consulting fee. GL-R’s and FC’s institutions received payment from the GSK group of companies for the performance of the clinical trial as well for execution of immune assays. NG and SD were employees of the GSK group of companies. NG owns stocks from GSK group of companies or stock options and has issued patents for AS01, AS03, and AS04.

## Members of the ECR-002 Study Group

Viviane Bechtold, Wivine Burny, Andrea Callegaro, Isabelle Carletti, Frédéric Clement, Sophie Delhaye, Arnaud Didierlaurent, Meral Esen, Laurence Fissette, Julian Gabor, Nathalie Garçon, Edwige Haelterman, Caroline Hervé, Yves Horsmans, Michel Janssens, Peter Kremsner, Geert Leroux-Roels, Arnaud Marchant, Philippe Moris, Tino F Schwarz, Fernanda Tavares da Silva, Pascale Van Belle, Pierre Van Damme, Robert A. van den Berg, Robbert van der Most, Dirk Zuchner.
