# Supplementary material for: Different Adjuvants Induce Common Innate Pathways That Are Associated with Enhanced Adaptive Responses against a Model Antigen in Humans
Source: Front Immunol. 2017 Aug 14;8:943. doi: 10.3389/fimmu.2017.00943 (PMC5557780; doi:10.3389/fimmu.2017.00943)
Supplement: Supplementary file 1 [file Table_1.docx]

**Table S1. Assessment of cytokine, chemokine and mRNA responses**

| **Cytokines and chemokines (by CBA in serum)** | **Genes/related genes of associated signaling pathway^#^** | **Selection rationale (for cytokine//gene)** |
| --- | --- | --- |
| - | *Ki67/MKI67* | cell proliferation marker |
| - | *Mx1* | IFN type I (antiviral innate cytokines) |
| - | *IL12A* | NK-cell and T-cell activation |
| IL-1β | *IL1B* | pro-inflammatory cytokine |
|  | *PTGS2/COX2* |  |
|  | *MKP1/DUSP14* |  |
| IL-6 | *NFATc2* | pro-inflammatory cytokine |
| TNF-α | *TNF* | pro-inflammatory cytokine |
|  | *TNFRSF9/41BB* |  |
|  | *FAS/TNFRSF6* |  |
| IFN-γ | *IFNG* | Th1 cytokine |
|  | *STAT1* |  |
|  | *IRF1* |  |
| IL-5 | *-* | Th2 cytokine |
| IL-10 | *-* | pro-inflammatory/regulatory cytokine |
| IP-10 | *CXCL10/IP10* | pro-inflammatory chemokine |
| MCP-1 | *-* | chemokine regulating monocyte/macrophage migration |

^#^ Expression of these genes was measured by whole blood qPCR. CBA, cytometric bead array. IFN-γ: Interferon γ. IL-1β/10/5/6: Interleukin-1β/10/5/6. IP-10: Interferon γ-inducible protein 10. MCP-1: Monocyte chemotactic protein 1. TNF-α: Tumor necrosis factor α. *TNFRSF9:* Tumor necrosis factor receptor superfamily member 9. *FAF1:* Fas associated factor 1. *STAT1:* Signal transducer and activator of transcription 1. *IRF1:* Interferon regulatory factor 1. *IFNG*: Interferon γ. *MX1:* Myxovirus (influenza virus) resistance 1. *CXCL10:* C-X-C motif chemokine ligand 10. *IL-12A:* Interleukin 12A. *IL-1β:* Interleukin-1 β. *PTGS2:* Prostaglandin-endoperoxide synthase 2. *DUSP1:* Dual specificity phosphatase 1. *NFATC2:* Nuclear factor of activated T-cells 2.
