# Supplementary material for: Different Adjuvants Induce Common Innate Pathways That Are Associated with Enhanced Adaptive Responses against a Model Antigen in Humans
Source: Front Immunol. 2017 Aug 14;8:943. doi: 10.3389/fimmu.2017.00943 (PMC5557780; doi:10.3389/fimmu.2017.00943)
Supplement: Supplementary file 2 [file Table_2.docx]

**Table S2. Associations of adaptive responses post-dose 2, with innate responses, reactogenicity and adaptive responses post-dose 1**

|  | **HBsAg-specific adaptive immune response at 2 weeks pII** | | | | | | | | | | | | | | | | | | | | | | | | | | | | | | | | |  |  |
| --- | --- | --- | --- | --- | --- | --- | --- | --- | --- | --- | --- | --- | --- | --- | --- | --- | --- | --- | --- | --- | --- | --- | --- | --- | --- | --- | --- | --- | --- | --- | --- | --- | --- | --- | --- |
|  | **Clinical laboratory and serum protein data** | | | | | | | | | | | | | |  | | **Gene expression data** | | | | | | | | | | | | | | | | | |  |
|  | **Y = CD40L^+^ CD4^+^ T cells** | | |  | | **Y = Antibodies** | | | | | | | |  | | **Y = CD40L^+^ CD4^+^ T cells** | | | | | | | |  | | | **Y = Antibodies** | | | | | | | | |
| β Input parameter | Estimate | SE | *P* value | | |  | | Estimate | | SE | | *P* value | | |  | | | | Estimate | | SE | | *P* value | | |  | | | Estimate | | SE | | *P* value | |  |
| Intercept (β_0_) | 1.51 | 0.18 | 8.8 E-15 | |  | | 0.65 | | 0.28 | | 0.02 | |  | | | | | 1.40 | | 0.32 | | 5.7 E-05 | | |  | | | -0.12 | | 0.52 | | 0.82 | | |  |
| Local reactogenicity pII | -0.02 | 0.02 | 0.37 | |  | | 0.02 | | 0.03 | | 0.45 | |  | | | | | -0.03 | | 0.04 | | 0.46 | | |  | | | 0.16 | | 0.06 | | **0.009** | | |  |
| Systemic reactogenicity pII | 0.01 | 0.01 | 0.31 | |  | | -0.01 | | 0.02 | | 0.51 | |  | | | | | 0.01 | | 0.03 | | 0.68 | | |  | | | -0.04 | | 0.04 | | 0.28 | | |  |
| Innate response pII; PC1 | -0.01 | 0.02 | 0.68 | |  | | 0.02 | | 0.03 | | 0.46 | |  | | | | | -0.14 | | 0.10 | | 0.16 | | |  | | | -0.09 | | 0.16 | | 0.57 | | |  |
| Innate response pII; PC2 | 0.05 | 0.04 | 0.21 | |  | | 0.08 | | 0.07 | | 0.23 | |  | | | | | 0.03 | | 0.11 | | 0.80 | | |  | | | -0.09 | | 0.18 | | 0.62 | | |  |
| Innate response pII; PC3 | -0.06 | 0.04 | 0.13 | |  | | 0.01 | | 0.06 | | 0.87 | |  | | | | | -0.10 | | 0.12 | | 0.38 | | |  | | | -0.30 | | 0.19 | | 0.11 | | |  |
| Ab responses pI^#^ | 0.10 | 0.03 | **0.004** | |  | | 0.43 | | 0.05 | | **1.6 E-13** | |  | | | | | -0.02 | | 0.06 | | 0.76 | | |  | | | 0.40 | | 0.09 | | **5.9 E-05** | | |  |
| CD4^+^ T-cell responses pI^*^ | 0.40 | 0.07 | **6.2 E-08** | |  | | 0.23 | | 0.11 | | **0.04** | |  | | | | | 0.54 | | 0.13 | | **0.0001** | | |  | | | 0.54 | | 0.21 | | **0.01** | | |  |
| AS01_B_ | 0.41 | 0.13 | **0.001** | |  | | 1.83 | | 0.19 | | **<2.0 E-16** | |  | | | | | 0.30 | | 0.23 | | 0.20 | | |  | | | 1.20 | | 0.36 | | **0.002** | | |  |
| AS01_E_ | 0.40 | 0.11 | **0.0003** | |  | | 1.84 | | 0.17 | | **<2.0 E-16** | |  | | | | | 0.34 | | 0.18 | | 0.07 | | |  | | | 1.52 | | 0.30 | | **2.9 E-06** | | |  |
| AS03 | 0.15 | 0.10 | 0.15 | |  | | 1.61 | | 0.16 | | **<2.0 E-16** | |  | | | | | 0.29 | | 0.17 | | 0.10 | | |  | | | 1.48 | | 0.27 | | **8.0 E-07** | | |  |
| AS04 | -0.04 | 0.10 | 0.71 | |  | | 0.78 | | 0.15 | | **2.6 E-07** | |  | | | | | -0.07 | | 0.16 | | 0.66 | | |  | | | 0.41 | | 0.25 | | 0.11 | | |  |

β, regression coëfficient. β_0,_ intercept for the Alum group (considered the baseline). pII, after the second injection. SE, standard error. ^#^HBs-specific antibody (Ab) responses pI were measured at 1 month post dose 1 (Day 30). *CD4^+^ T-cell responses pI were measured at 2 weeks post dose 2 (Day 14). P-values <0.05 are bolded.
