# Supplementary figures and images for: Different Adjuvants Induce Common Innate Pathways That Are Associated with Enhanced Adaptive Responses against a Model Antigen in Humans
Source: Front Immunol. 2017 Aug 14;8:943. doi: 10.3389/fimmu.2017.00943 (PMC5557780; doi:10.3389/fimmu.2017.00943)

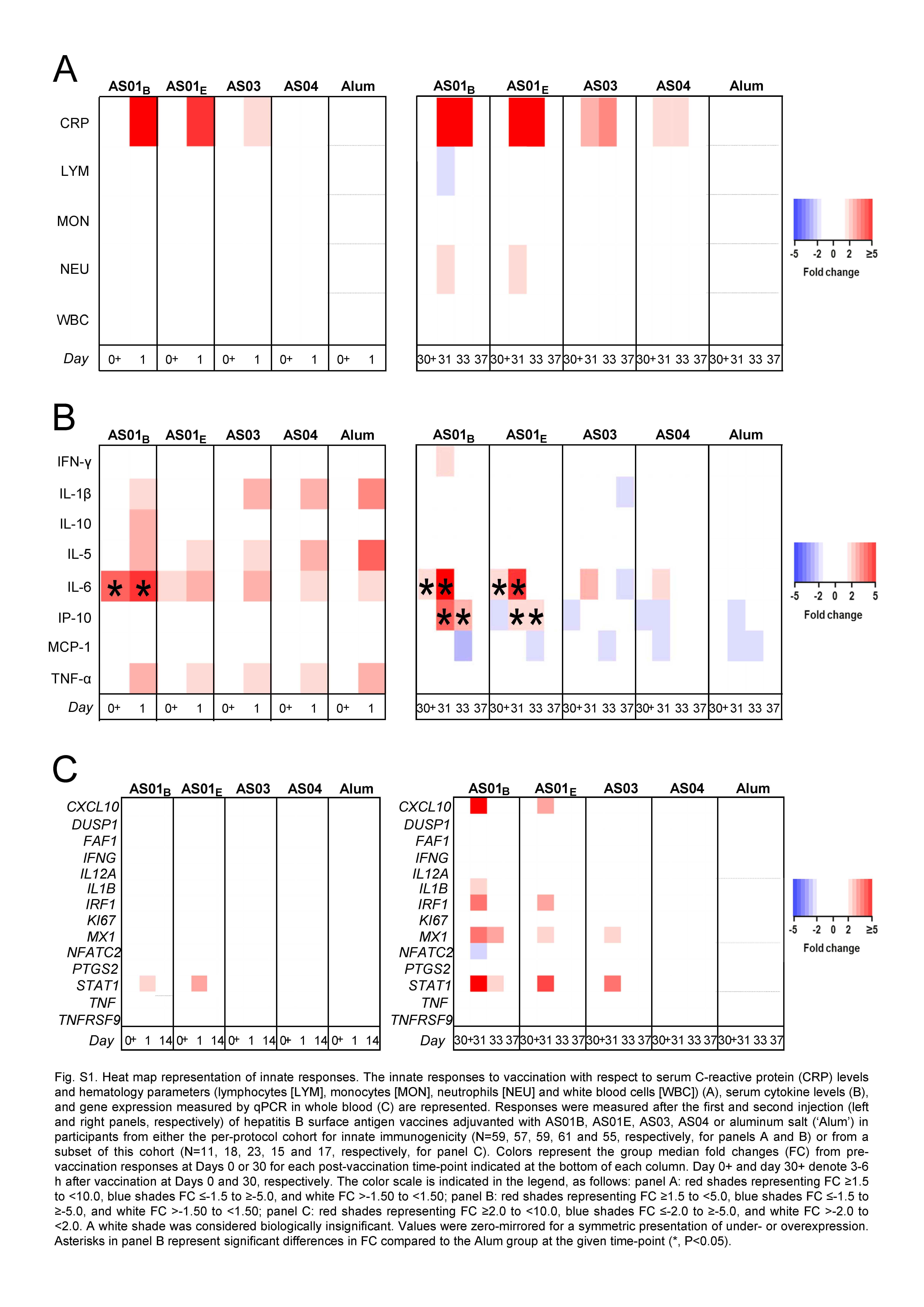

Supplement: Supplementary file 3 [file Image_1.TIF]

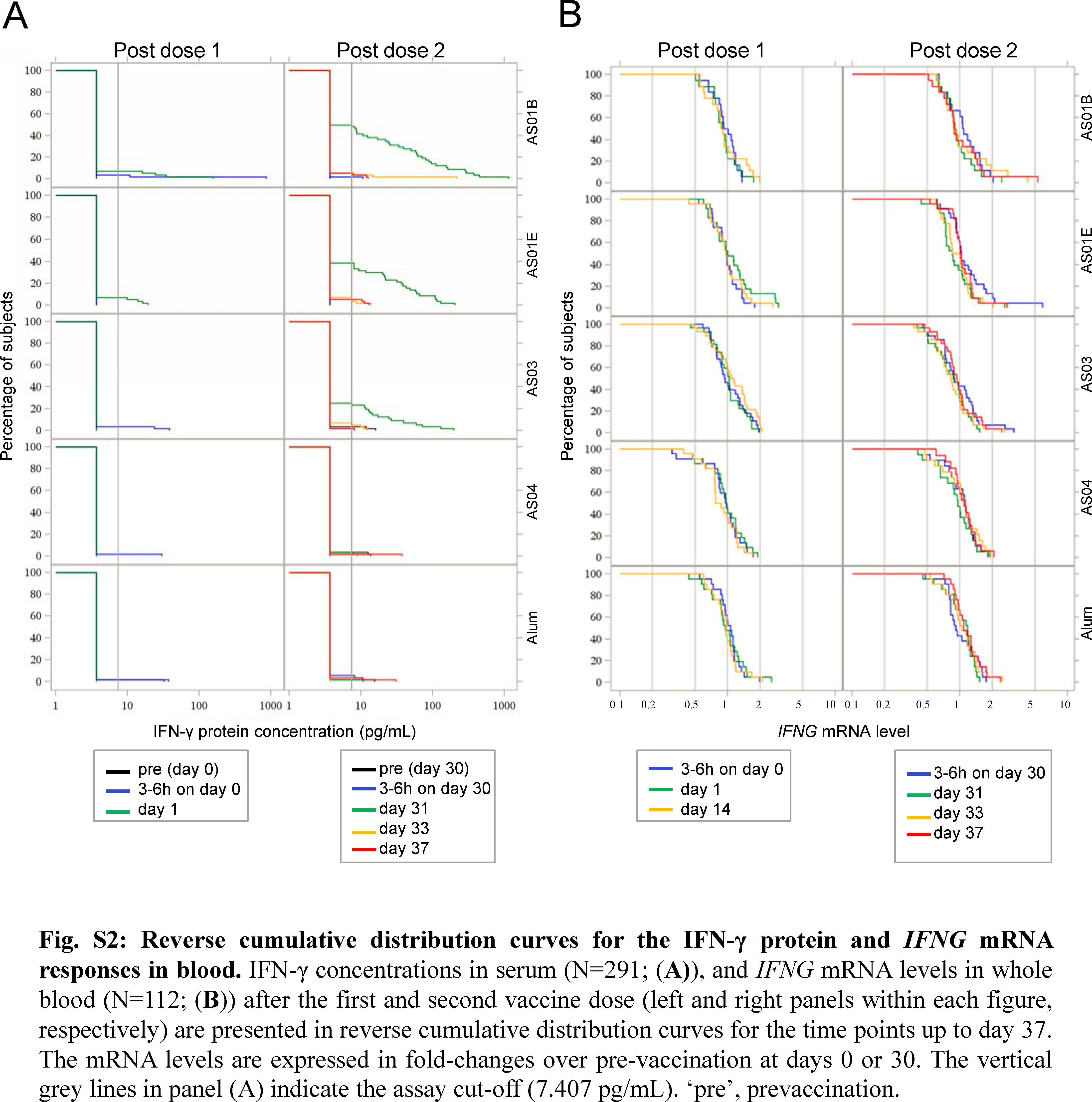

Supplement: Supplementary file 4 [file Image_2.TIF]

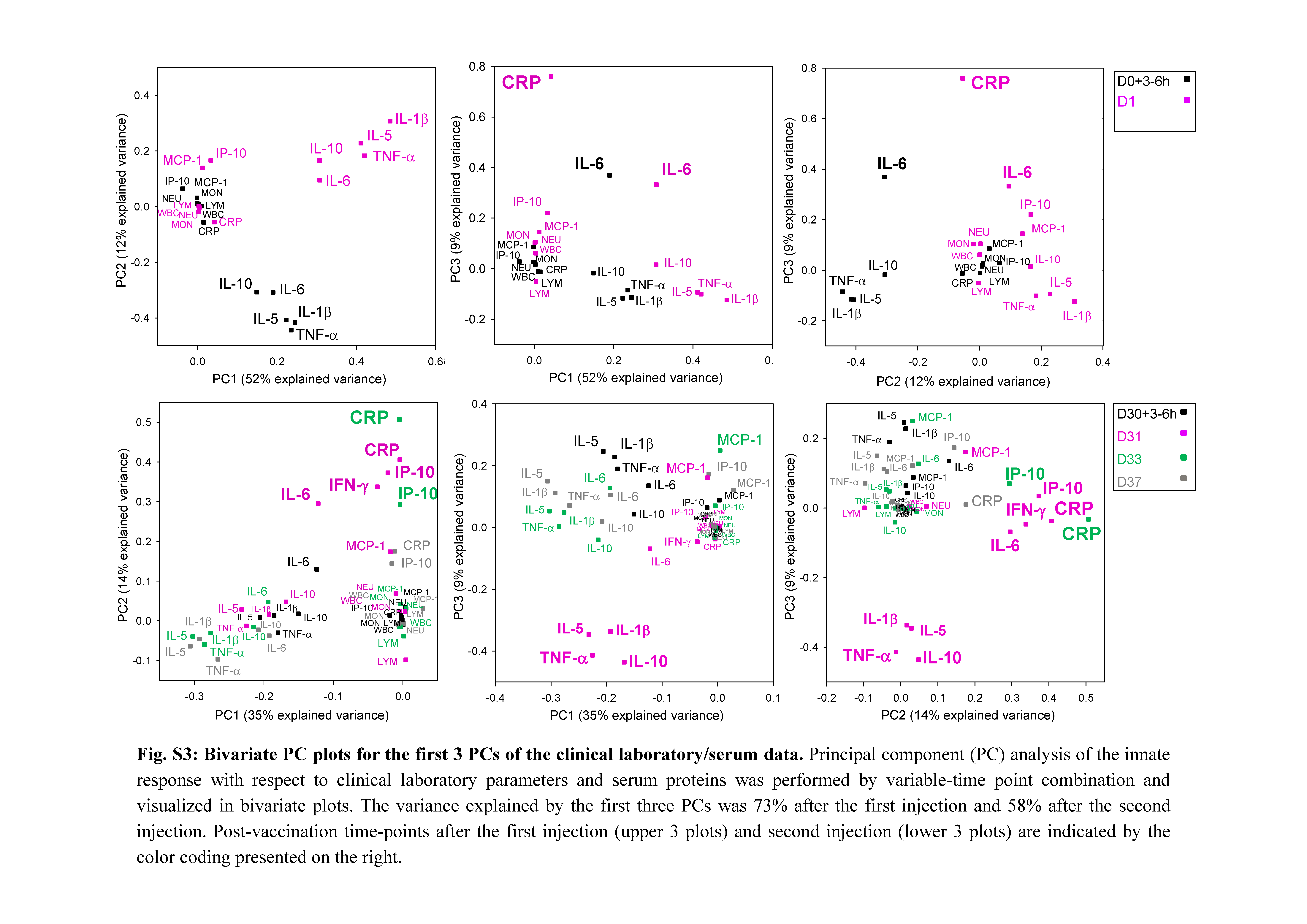

Supplement: Supplementary file 5 [file Image_3.TIF]

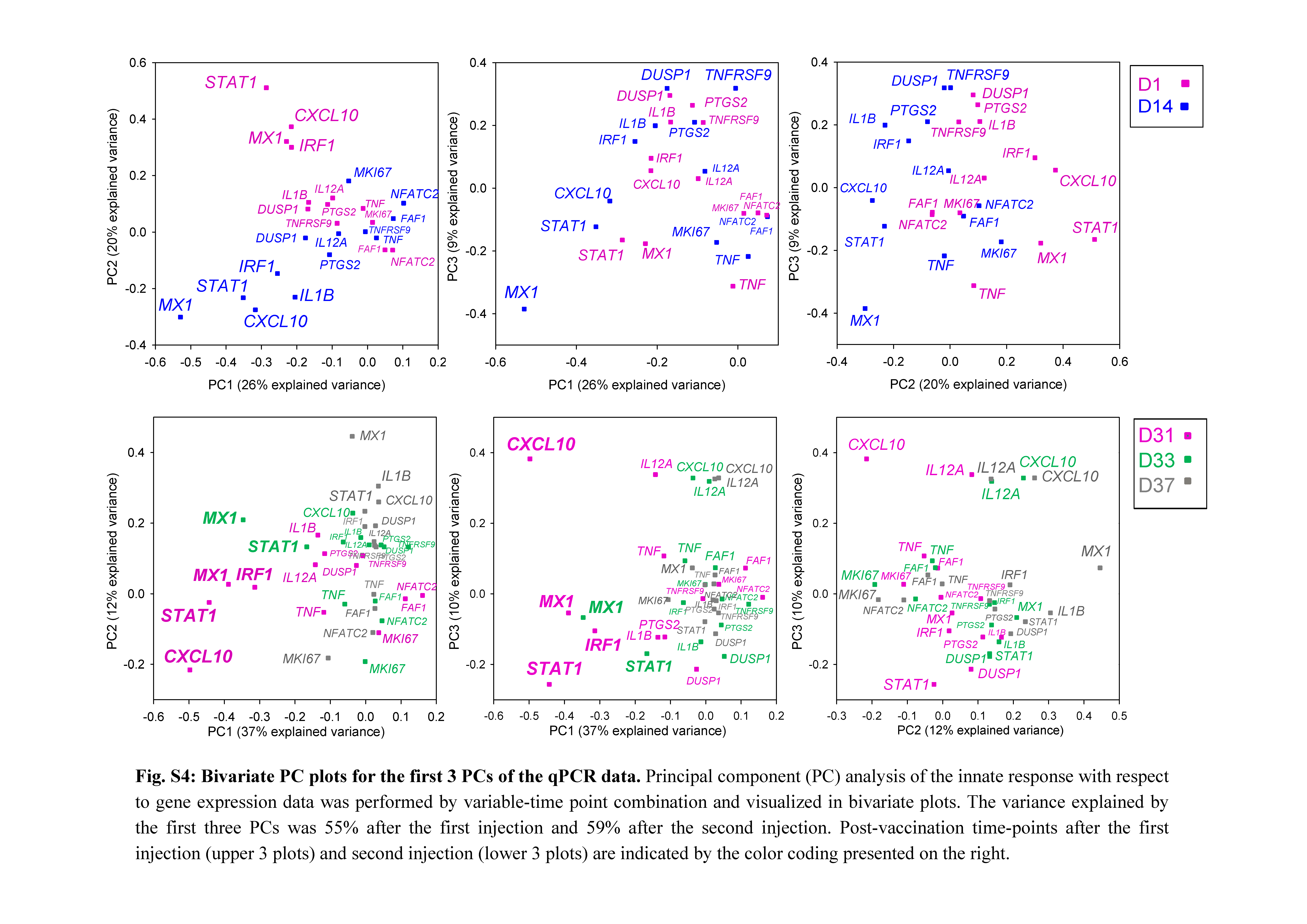

Supplement: Supplementary file 6 [file Image_4.TIF]
